# Actionable heterogeneity of hepatocellular carcinoma therapy-induced senescence

**DOI:** 10.1007/s00262-025-04060-w

**Published:** 2025-05-15

**Authors:** Pujan Engels, Andras Szolek, Sebastian Hörner, Georgios Vavouras Syrigos, Kim Hebbel, Michelle Schmidtke, Min Zhou, Maria Mateo-Tortola, Caroline Schönfeld, Sylwia Anna Stefanczyk, Katharina Wolter, Sepideh Babaei, Michael Schindler, Manfred Claassen, Daniel Dauch, Lars Zender, Ana Tapía-Abellán, Alexander N. R. Weber

**Affiliations:** 1 Department of Innate Immunity, Institute of Immunology, University of Tübingen, Tübingen, Germany; 2 iFIT Cluster of Excellence 2180, ‘Image-Guided and Functionally Instructed Tumor Therapies’, University of Tübingen, Tübingen, Germany; 3 Clinical Collaboration Unit Translational Immunology, German Cancer Consortium (DKTK), Department of Internal Medicine, University Hospital Tübingen, Tübingen, Germany; 4 Department of Medical Virology, Institute for Medical Virology and Epidemiology of Viral Diseases, University Hospital Tübingen, Tübingen, Germany; 5 DZIF Partner Site Tübingen, Tübingen, Germany; 6 Department of Medical Oncology and Pneumology, University Hospital Tübingen, Tübingen, Germany; 7 German Cancer Research Consortium (DKTK), Partner Site Tübingen, German Cancer Research Center (DKFZ), 69120 Heidelberg, Germany; 8 Department of Internal Medicine I, University Hospital Tübingen, Tübingen, Germany; 9 Department of Computer Science, University of Tübingen, Tübingen, Germany; 10 M3 Research Center, University Hospital Tübingen, Tübingen, Germany; 11 Institute for Bioinformatics and Medical Informatics, University of Tübingen, Tübingen, Germany; 12 Cluster of Excellence 2124 CMFI, Controlling Microbes to Fight Infection, University of Tübingen, Tübingen, Germany

**Keywords:** Senescence, Hepatocellular carcinoma, Immunotherapy, Immunology

## Abstract

Therapy-induced senescence (TIS) is a stable cell cycle arrest in cancerous cells favoring immune control upon immune cell recruitment and activation via a senescence-associated secretory phenotype (SASP). Numerous studies have investigated the therapeutic applicability of TIS in hepatocellular carcinoma (HCC), a frequent cancer with high morbidity and mortality. Despite these efforts, a comprehensive understanding of how TIS may expose vulnerabilities specifically for immunotherapies, a potent means of cancer therapy, in HCC remains incomplete. Therefore, we conducted systematic studies to carefully characterize actionable and shared SASP- or other senescence-associated molecular parameters of TIS. We systematically compared the TIS inducers, etoposide and alisertib with a novel TIS inducer, CX5461, for their effects on SASP, surfaceome and innate immune clearance of representative human HCC cell lines. Surprisingly, all three compounds induced both metastasis surface antigens but also immunotherapeutically tractable antigens like CD95 (Fas), CD276 (B7-H3) and CD340 (Her2). This was verified in four representative HCC cell lines and publicly available datasets of HCC. Interestingly, alisertib, etoposide and CX5461 rendered senescent HCC vulnerable to be targeted by either T-cell-engaging bispecific antibodies or CAR NK cells. Collectively, our study indicates that heterogenous, but selective features of HCC senescence may be exploited by different immunotherapeutic approaches.

**Supplementary Information:**

The online version contains supplementary material available at 10.1007/s00262-025-04060-w.

## Introduction

Hepatocellular carcinoma (HCC), is considered the most frequent primary liver malignancy and the fourth leading cause of cancer-related mortality world-wide [1, 2]. Despite progress in terms of molecular understanding and novel therapies (e.g., targeted and immunotherapies), 5-year survival is still around 18%. Cellular senescence is considered a critical cellular state in HCC, with relevance both for progression as well as treatment [3, 4]. Senescence is defined via a stable cell-cycle arrest and can be induced in multiple ways, including telomere shortening (replicative senescence), oncogene activation, oxidative damage or treatments/therapies such as radiation or chemotherapy (therapy-induced senescence, TIS) [5, 6]. These pathways often converge on a DNA damage response, which may include cytoplasmic leakage of DNA. The expression of p21^CIP1^ (coded by CDKN1A) and p16^INK4a^ (encoded by CDKN2A) as cell cycle suppressors prevents progression from S phase to G1 phase. This halts further proliferation of these pre-malignant cells and is a molecular hallmark of the senescent state. Concomitantly, an increase in cell size, metabolic reprogramming and the accumulation of senescence-associated (SA) β-galactosidase (SA β-gal) are observed [5, 6]. Moreover, a complex senescence-associated secretory phenotype (SASP) is induced and triggers mechanisms of immune-mediated removal by attracting immune cells through cytokines and chemokines. This may result in immune-mediated clearance of pre-malignant senescent cells [6–8]. On the other hand, senescent cells remain viable and metabolically active so that they represent a continuous threat for tumorigenesis in case additional mutations are acquired that re-start cell proliferation when intrinsic immune-clearance is insufficient. Moreover, released matrix modifiers, growth factors, chemokines, cytokines may favor metastasis and/or adversely affect the tumor microenvironment [6]. Despite many clear general hallmarks, a great degree of heterogeneity in terms of these different anti- or pro-tumorigenic effects has been appreciated between different tumor types and types of senescence induction even among different TIS regimens [6, 9]. What is clear, however, is that TIS may not only provide a temporal window of opportunity in which potentially oncogenic cells may be removed intrinsically by the immune system or by general therapeutic intervention; rather specific types of TIS may also be combined with matching so-called senolytic approaches [7, 10]. The most widely used senolytics are the small molecule inhibitors navitoclax, quercetin, dasatinib and fisetin. While navitoclax targets anti-apoptotic Bcl-2 proteins, dasatinib is a potent SRC and ABL kinase inhibitor [11, 12]. However, their main mechanism of action is still not fully understood. An additional example is senolytic chimeric antigen receptor (CAR) T cells directed against the senescence-associated surface antigen, uPAR [13]. Altered surface antigens could also be exploited using receptor agonists or antagonists, or antibody-dependent cellular cytotoxicity, a concept used successfully for decades in lymphoma therapy [14]. Thus, TIS-induced changes to intracellular pathways and surface antigens may provide novel means of interventions, provided actionable hallmarks of specific TIS regimens can be identified.

To characterize heterogeneity in HCC TIS and to explore exposure of shared actionable vulnerabilities, we initially characterized the effects of three TIS inducers – alisertib (an Aurora kinase A inhibitor), etoposide (a topoisomerase II inhibitor), and CX5461, an RNA polymerase I inhibitor – on well-established HCC cell lines with different p53 mutational status, systematically comparing changes in transcription and intracellular innate pathways, SASP characteristics and the senescence-associated surfaceome (SASome). Interestingly, all TIS inducers caused metastasis-associated surfaceome changes but also TIS inducer-specific alterations. Selected alterations were actionable via death receptor ligands, therapeutic antibodies, or NK cell chimeric antigen receptor (CAR) therapy in vitro. Collectively, our work provides a rationale for exploring HCC senescence heterogeneity for the identification and exploitation of specific combinations for a given TIS inducer with a matched immunotherapeutic/senolytic approach in HCC.

## Materials and methods

### Reagents

Senescence inducers alisertib (S1133) and CX5461 (S2684) were from Selleckchem and etoposide from Sigma-Aldrich (E1383). The stimulant 2′3′-cGAMP (tlrl-nacga23-02) and inhibitor H-151 (inh-h151) were acquired from Invivogen. Recombinant human sFas Ligand was from Peprotech (310-03H) and activating anti-Fas antibody CH11 from Sigma-Aldrich (05-201). Antibodies used for fluorescence microscopy and immunoblotting are listed in Table S3.

### HCC cell line culture and senescence induction

HUH7, HLE and HLF cells (kindly provided by Daniel Dauch, University Hospital Tübingen) were cultured and stimulated in complete DMEM medium (Sigma, D5796-24X500ML) supplemented with 1% L-glutamine, 1% Sodium pyruvate and 1% non-essential amino acids. HepG2 cells (ATCC, HB-8065) were grown and stimulated in complete RPMI medium (Sigma, R8758-24X500ML) Senescence was induced using 1 μM Alisertib (Selleckchem, S1133), 500 nM CX5461 (Selleckchem, S2684) or 10 μM Etoposide (Sigma-Aldrich, E1383) diluted in respective cell culture medium. Cells treated with Alisertib or Etoposide were stimulated for 48 h or 72 h at 37 °C and 5% CO_2_. CX5461-treated cells were incubated for 24 h and rested for 48 h or 72 h at 37 °C and 5% CO_2_.

### Flow cytometry

Cells were detached using PBS, 5 mM EDTA and filtered through a cell strainer. 200 μL of cells were transferred into a 96-well plate (U-bottom) and centrifuged for 5 min at 500 × *g*. Afterward, blocking was performed using pooled human serum diluted 1:10 in cell staining buffer (Biolegend, 420,201) for 20 min at RT. After washing, the samples were stained for 30 min at RT in the dark, repeatedly washed and fixed with fixation buffer. Fluorescent detection of β-galactosidase hydrolysis was performed according to the manufacurer’s protocol (Thermo Fisher, C10841). After repeated washing for 10 min at RT in the dark, the cell pellets were resuspended in 100 µL PBS and measurements were performed on a FACS Canto II or LSR Fortessa (BD Bioscience, Diva software). Analysis was performed using FlowJo V10 analysis software.

### Fluorescence microscopy of fixed human HCC cells

4 × 10^4^ HCC cells were seeded in a 24-well plate containing 12 mm coverslips. After senescence induction (as described above) cells were washed with PBS and fixed with fixation buffer (Biolegend, 420,801) for 10 min at RT in the dark. Afterward cells were washed again with PBS and blocked/permeabilized with permeabilization buffer containing 0.1% Triton X-100, 0.1% Tween 20 and 5% bovine serum albumin. Cells were then incubated with primary antibody (Table S3) for 1 h in staining buffer containing 0.1% Tween 20 and 5% bovine serum albumin. Afterward cells were washed three times with staining buffer and incubated with the secondary antibodies (Table S3). After further washing, cells were incubated with Hoechst 33342 (Thermo Fisher; 1 μg/mL) for 5 min to stain nuclear DNA. Finally, coverslips were mounted (ProLong™ Diamond Antifade Mountant, 815 Thermo Fisher, P36961) on glass slides and left to dry overnight at RT in the dark. Microscopy was performed using the Zeiss LSM800 Confocal microscope (AiryScan mode), and images were analyzed using ImageJ-Win64 and ZEN Blue3 software.

### ELISA

To measure cytokine release, ELISA Kits for hIL-8 (Biolegend, 431504), hCXCL10 (Biolegend, 439904), VEGF-A (Biolegend, 446504), MMP9 (R&D systems, DY911-05) and MPO (R & D systems, DY3174) were used according to the manufacturer’s instructions. Samples were assessed in triplicates.

### Senescence-associated β-galactosidase (SA-β-Gal) detection

Fluorimetric detection of SA-β-Gal was performed according to manufacturer’s instructions using a β**-**Galactosidase Detection Kit (Abcam, ab176721). In brief, target cells were seeded on a 6-well plate and senescence was induced as described above. Afterward, cells were washed, trypsinized and counted. 1.5 × 10^5^ cells were centrifuged for 5 min at 500 × *g* and resuspended in 150 µl lysis buffer containing 0.1% β-mercaptoethanol. After distributing lysates into 96-well black plates (SPL Life Sciences, 33,396), working solution was added containing fluorescein-digalactoside (FDG) and lysates were incubated at 37 °C for 1 h. Fluorescence intensity was measured with a BMG Labtech Fluostar Optima microplate reader at Ex/Em = 490/525 nm.

### Immunoblotting

For analysis of protein expression by immunoblotting whole cell lysates were generated in RIPA buffer containing protease inhibitor (Sigma-Aldrich, 11836153001). Afterward, protein concentrations were determined by Bradford assay. Whole cell lysates were mixed with LDS sample buffer (Invitrogen, NP0008) as well as reducing agent (Invitrogen, NP0009), denatured by boiling for 5 min at 95 °C and samples were subjected to electrophoresis on 8–12% acrylamide gels. After running for 120 min at 120 V, samples were transferred to a 0.45 µm nitrocellulose membrane in a semi-dry transfer for 40–45 min. The membrane was blocked with 5% bovine serum albumin (BSA) (w/v) in Tris-buffered saline solution with 0.1% (v/v) Tween-20 (TBS-T) for 1 h at RT and incubated overnight at 4 °C in 5 ml buffer containing primary antibody (Table S3). Afterward, membranes were washed with TBS-T and incubated with secondary, HRP-conjugated antibodies (Table S3), diluted in TBS-T containing 5% BSA. After 1 h incubation, membranes were washed with TBS-T and ECL substrate was added according to the manufacturer’s instructions to detect chemiluminescence. Imaging was performed using Licor camera Odyssey Imaging system in the chemiluminescence channel, and pictures were analyzed and edited in Image StudioTM Lite software.

### NK92 MI cell culture and killing assay

NK92 MI cells (kindly provided by Melanie Märklin, University Hospital Tübingen) were cultured in complete IMDM medium (Lonza, 12-722F). To investigate NK cell-mediated cytotoxicity target cells were seeded in a 24-well plate and senescence was induced as described above. Afterward, target cells were washed and NK92 MI cells were added in different effector to target ratios. Standard and maximum release were analyzed for normalization to respective target cell numbers and NK cell independent cell death, respectively. After 3 h incubation at 37 °C and 5% CO_2_ supernatants were harvested and centrifuged for 5 min at 500 × *g* to remove cell debris. Subsequently, cell death was determined using a cytotoxicity detection kit (Roche, 11,644,793,001).

### NK92 and CAR-NK cell culture and killing assay

NK92 and CAR NK92 cells (kindly provided by Guillermo Urena Bailen, University Hospital Tübingen) were cultured in complete MEMα GlutaMax medium supplemented with 1000 U/µl IL-2. For investigation of NK cell-mediated cytotoxicity a calcein acetoxymethyl esther (calcein AM) assay was performed (Biolegend, 425201). Prior to the calcein AM assay, target cells were seeded in a 24-well plate and senescence was induced as described above. Calcein was reconstituted in DMSO to a stock concentration of 1 µg/µl. and added to live cell imaging solution (Invitrogen, 12,363,603) at a ratio of 10 µl/ml. Cells were incubated with calcein AM for 1 h at 37 °C and 5% CO_2_. Afterward, target cells were washed and NK92 MI cells were added in different effector to target ratios. Standard and maximum release were analyzed for normalization to respective target cell numbers and NK cell independent cell death, respectively. After 3 h incubation at 37 °C and 5% CO_2_ supernatants were harvested and centrifuged for 5 min at 500 × *g* to remove cell debris. Subsequently, supernatants were transferred to black 96-well plates and measured at 488 nm/520 nm.

#### Neutrophil co-culture and transwell assay

Primary human neutrophils were isolated as described [1]. After isolation, purity and activation was analyzed by flow cytometry as described in [2]. One day prior to isolation 1.5 × 10^5^ senescent and non-senescent HUH7 cells were seeded in a 24-well plate. Freshly isolated neutrophils were added to transwells and cell culture inserts (Thermo Fisher, 141006) were transferred into respective HUH7-containing wells. To investigate SASP-mediated migration, media of the lower layer was exchanged to conditioned or fresh HUH7 media. Migrated primary human neutrophils were counted after 6 h.

To determine neutrophil activation after HCC cell co-culture, 2 × 10^5^–4 × 10^5^ HUH7 cells were seeded in a 6-well plate and senescence was induced (as described above). Subsequently, HUH7 were washed, and 1.5 × 10^5^ freshly isolated neutrophils were added in complete RPMI media. After 3 h incubation at 37 °C and 5% CO_2_, supernatants were harvested and neutrophil activation was determined by MPO and MMP9 ELISA.

#### LegendScreen of HCC cell lines

To characterize the surfaceome of senescent HCC cell lines, a previous protocol [15, 16] was adapted. In brief, 1.5 × 10^6^–2.5 × 10^6^ HUH7 or HepG2 cells were seeded on four T175 flasks per condition. After senescence induction (as described above) cells were detached using PBS + 5 mM EDTA (Thermo Fisher, 15575020) and filtered through a cell strainer. LegendScreen was performed according to manufacturer’s instruction using the LegendScreen human PE kit (BioLegend, 700,007). Measurements were performed on a MACSQuant Analyzer 10 (Miltenyi Biotec), and data were analyzed using FlowJo V10 analysis software.

#### Fas-mediated cytotoxicity assays

To initiate Fas-mediated apoptosis in senescent HCC cell lines, senescence was induced as described above and cells were treated with 150 ng/ml soluble Fas ligand (Preprotech, 310-03H) or different concentrations of activating Fas antibody CH11 (Sigma-Aldrich, 05–201), respectively. After incubation for 6 or 24 h at 37 °C and 5% CO_2_, cytotoxicity was determined using the cell counting kit-8 (Dojindo Laboratories, CK04-13) and relative cell death was determined by normalizing absorbance values of Fas ligand- and CH11-treated conditions to untreated controls.

#### Bispecific antibody assays

To investigate senolytic capacities of the B7-H3xCD3 bi-specific antibody CC-3 [17], target cells were seeded in a 24-well plate and senescence was induced (as described above). Subsequently, target cells were cultured with monocyte-depleted PBMC of healthy donors (E:T 2:1) in the presence or absence of B7-H3xCD3 or MOPCxCD3. To test concentration dependency, different dilutions of the bi-specific antibodies were tested (0.0005–5 nM). For analysis of T cell activation, CD69 expression of CD3 positive cells was determined after 24 h. Furthermore, supernatants were collected and analyzed for IFNγ by ELISA (as described above).

#### Transcriptome data analysis

RNA sequencing data were processed with the nf-core/rnaseq version 1.4.2 pipeline using its STAR genome alignment subpath. Differential expression analysis was performed with PyDESeq2 using read count data from featureCounts, aggregated by HGNC symbol. Log fold-changes and differential expression p values were calculated for each gene under each treatment in each cell line between 3 passages of untreated samples and 2 × 3 passages of treated samples at two timepoints.

Gene set enrichment was performed using the preranked GSEA method on the Broad Institute’sh ‘hallmark’ gene sets, with log fold-changes serving as the ranking metric. Raw enrichment scores (ES) were converted to z-scores by estimating the mean and standard deviation of the ES null distribution for every gene set by bootstrapping (1000 random gene sets of the same size).

Transcriptome data were deposited in the Gene Expression Omnibus under ID [data submission started; ID to be added in proof].

#### Surfacesome data analysis

The median fluorescent intensity (MFI) of each surface antigen was normalized against the baseline of the corresponding isotype control’s MFI. For large (*n* > 40) isotype sets on a plate, in order to minimize the effect of the isotype control’s MFI variability propagating to a large number of markers, the baseline MFI was determined as the mode of a Gaussian kernel density estimator (bandwidth = 0.05). Differential expression was defined as the fold change of the normalized marker MFIs between the treated and untreated conditions.

Markers displaying bimodality were identified in a conservative manner, without relying on parametric assumptions about the underlying distributions. For every marker, the quantile density function (QDF) of fluorescent intensity was computed as the derivative of the quantile function smoothed with a 5 percentile wide quadratic Savitzky-Golay window filter. These QDFs were normalized against a reference function tracking the 95th percentile of all 2984 QDFs. If the normalized ratio exceeded 2 at any point for a marker, it was flagged as bimodal, and the location of the maximum was reported as the separating quantile between the low- and high-intensity cell populations.

#### Statistical analysis

Experimental data were analyzed using Excel 2019 (Microsoft) and/or GraphPad Prism 8 and 9, microscopy data with ImageJ-Win64 or ZenBlue3 software, flow cytometry data with FlowJo V10. Normal distribution in each group was always tested using the Shapiro–Wilk test first for the subsequent choice of a parametric (ANOVA, Student’s t-test for normally distributed data) or non-parametric (Kruskal–Wallis) test as indicated in figure legends. P values (*α* = 0.05) were then calculated, and multiple testing was corrected for in Prism, as indicated in the figure legends. Comparisons made to unstimulated control, unless indicated otherwise, were denoted by brackets.

## Results

### Innate immune responses are influenced by senescence and SASP heterogeneity

**Fig. 1:**
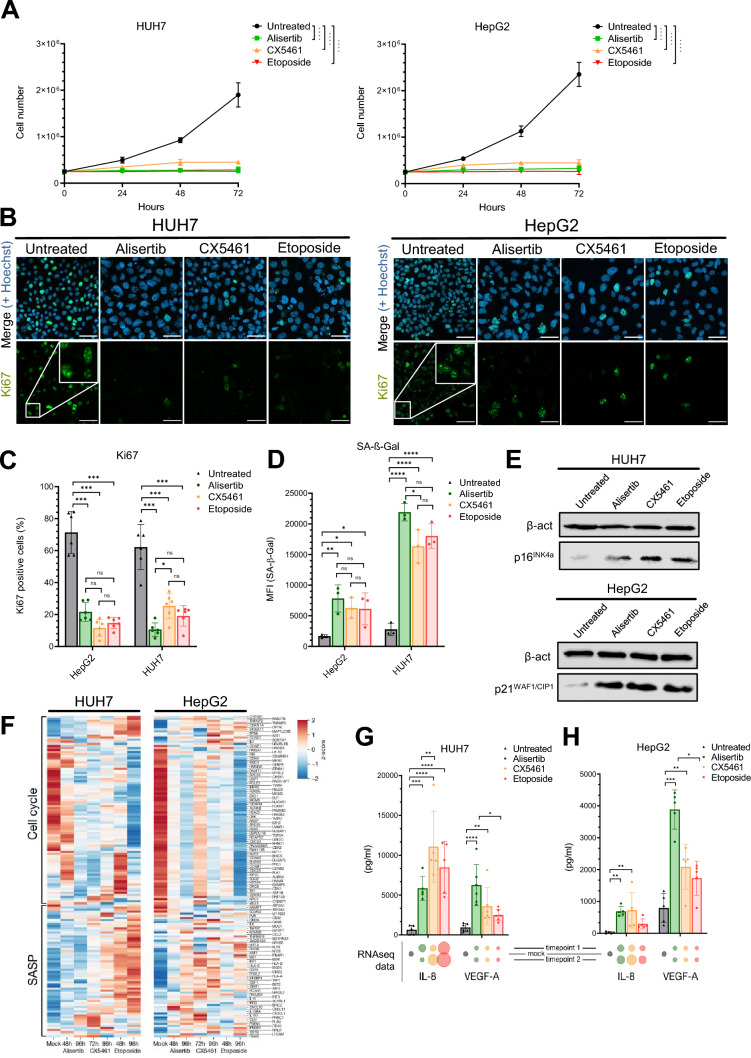
Comparable TIS induction by alisertib, CX5461 and etoposide in HUH7 and HepG2 HCC cell lines. **A** Cell number quantified in technical triplicates at the indicated time points (*n* = 3, representative data, mean + SD, ns *p* > 0.05 not indicated, *p < 0.05, **p < 0.01, ***p < 0.001 ****p < 0.0001 according to two-way ANOVA). **B**, **C** Ki67 immunofluorescence after treatment with 1 μM Alisertib (72 h), 500 nM CX5461 (96 h) or 10 μM Etoposide (72 h). CX5461-treated cells were stimulated for 24 h and rested for 72 h, quantified in C, (*n* = 3, each dot represents one 3 × 3 tile, 2 tiles per experiment, combined data, mean + SD, ns *p* > 0.05, *p < 0.05, **p < 0.01, ***p < 0.001 ****p < 0.0001 according to two-way ANOVA). **D** Fluorimetric detection of SA-β-Gal after treatment with 1 μM Alisertib (72 h), 500 nM CX5461 (96 h) or 10 μM Etoposide (72 h). CX5461-treated cells were stimulated for 24 h and rested for 72 h (*n* = 3 biological replicates, combined data, mean + SD, ns *p* > 0.05, *p < 0.05, **p < 0.01, ***p < 0.001 ****p < 0.0001 according to two-way ANOVA) **E** p21WAF/CIP1 and p16INK4a immunoblot analysis after treatment with TIS inducers (*n* = 3 biological replicates, representative data). **F** Heat-map indicating senescence-associated transcriptional changes upon treatment of HCC cell lines with senescent inducers (*n* = 3 biological replicates, combined data). **G**, **H** IL-8 and VEGF secretion from (above) and mRNA regulation (below) in senescent HUH7 (**G**) and HepG2 (**H**) cell lines analyzed by triplicate ELISA or RNAseq, respectively (*n* = 5 biological replicates, combined data, mean + SD. ns *p* > 0.05 not indicated, *p < 0.05, **p < 0.01, ***p < 0.001 ****p < 0.0001, according to Kruskal–Wallis test). Normalized RNAseq expression levels relative to the untreated condition are indicated below, with dot areas proportional to fold change

In order to study similarities and differences in HCC-TIS, we induced senescence in the well-known HCC cell lines, HUH7 (p53-mutated) and HepG2 (p53 wild-type). Assessment of stable growth arrest via cell counting (Fig. 1A), Ki67 staining (Fig. 1B, quantified in C), and SA-β-Gal induction via fluorimetric assay (Fig. 1D) confirmed successful TIS induction by alisertib, CX5461 and etoposide. This was further supported by p21^WAF/CIP1^ induction in HepG2 and p16^INK4a^ induction in HUH7 cells, respectively (Fig. 1E). Differences between treatments were largely statistically non-significant. Moreover, we monitored transcriptional changes and found typical senescence hallmarks proposed by Ruscetti et al. [18] to clearly be evident in these cultures (Fig. 1F). Collectively, these data show that all three TIS inducers led to a state of senescence that was highly comparable regarding the most accepted general hallmarks of senescence.

However, despite these common hallmarks, we also noted considerable heterogeneity on a transcriptomics level (Fig. 1F), giving rise to the notion that there might be informative treatment-specific differences. Differences were also visible at the level of certain chemokines and cytokines. To check if these were reflected at the protein level, we assessed the SASP chemokines IL-8, CXCL10 and the growth factor VEGF and confirmed differences between TIS treatments, whereas CX5461 induced IL-8 and CXCL10 secretion most highly, alisertib was the strongest VEGF inducer (Fig. 1G, H, S1A). Aware of the effect of chemokines on neutrophils and NK cells as mediators of senescence immunosurveillance [18–20], we also investigated whether these differences between TIS inducers affected the response of neutrophils and NK cells on senescent HUH7 and HepG2 cells. Indeed, neutrophil migration (Fig. S1B) was found to differ and was only increased for CX5461 and etoposide (Fig. S1C). Moreover, these effects were elevated in the presence of SASP conditioned media taken from senescent HCC cells 24 h after senescence induction. When the activation of neutrophils was assessed by myeloperoxidase (MPO) and matrix metalloprotease (MMP)-9 release, a strong response was observed for CX5461 and Etoposide (Fig. S1D). Of note, the effects were independent of the TIS inducers themselves, as these had been removed by extensive washing prior to collection of HCC cell line supernatants or co-incubation. Next, we investigated the effect on NK cells by assessing the ability of the NK-92 MI cell line to lyse senescent HCC cells (Fig. S1E). In line with the previous results, senescence induction generally increased killing moderately [18, 19], but CX5461 was significantly more effective in promoting cell lysis in both HUH7 and HepG2 cells than other inducers (Fig. S1F). However, IMR90 fibroblasts, another non-HCC model of senescence (most frequently used for oncogene-induced senescence), showed the opposite effects, suggesting the outcome in HCC cell lines to be cell-type specific (Fig. S1G). Collectively, our data for these HCC cell lines show that despite a consistent senescent hallmark, there is considerable heterogeneity between TIS inducers that may be functionally relevant for specific SASP components and the response of innate immune cells.

### HCC therapy-induced senescence increases a metastasis-associated surfaceome

**Fig. 2:**
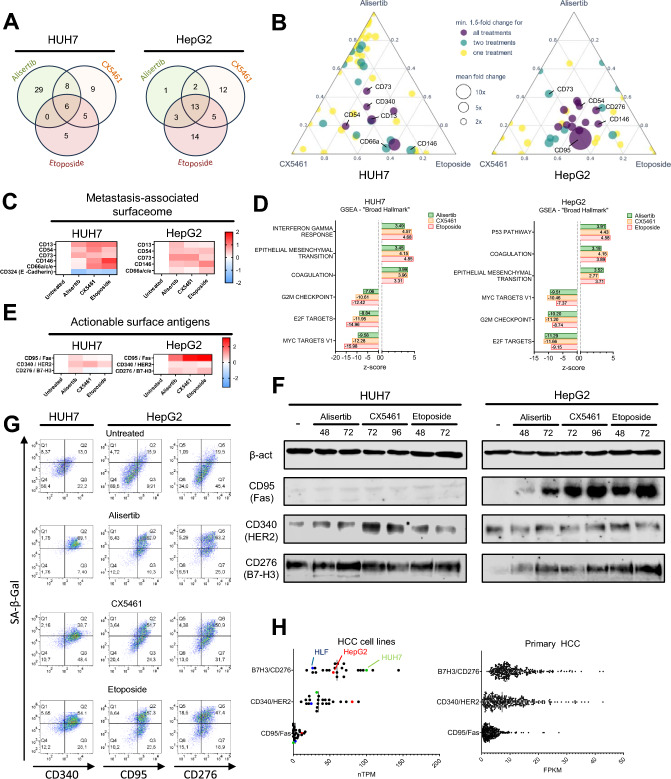
Characterizing the senescent HCC surfaceome. **A** Venn diagram indicating upregulated markers determined by SASome analysis, with an inclusion threshold of ≥ 1.5 fold change under at least one treatment compared to untreated surface expression. **B** Ternary plot showing the magnitude of upregulation of the same surface antigens under the three senescence inducing treatments. Marker size is proportional to average fold change, with marker position reflecting the relative contribution of each treatment to said average. **C** Expression changes of metastasis-associated surface antigens. **D** Gene set enrichment using the pre-ranked GSEA method on the Broad Institute’s Hallmark gene sets indicating the top 3 enriched gene sets expression (*n* = 3 biological replicates)**. E** Expression changes of pre-determined actionable surface antigens. **F** Immunoblot validation of surface marker expression (*n* = 3 biological replicates, representative data). **G** Flow cytometric validation of surface marker and SA-β-Gal expression in HCC cell lines, with reciprocal correlation analysis between SA-β-Gal and surface marker expression (*n* = 3 biological replicates, representative data). **H** CD276, CD340 and CD95 expression in HCC cell lines and primary HCC patient samples. Data derived from publicly available RNAseq dataset (Human Protein Atlas) of 24 HCC cell lines and 365 patient samples

It is well known that NK cell killing is mediated by an integration of signals based on cell surface molecules sensed on target cells [21, 22]. Based on the differences observed before, we speculated that alisertib, CX5461 and etoposide would differentially affect the surfaceome of the HCC cell lines. To explore this, we tested the surface expression of CD54/ICAM-1, a previously reported modulator of NK cell-target cell interaction [23]. In line with the strong effect on NK cell killing induced by CX5461, we noted a pronounced increase of ICAM-1 expression on CX5461-treated HUH7 cells (Fig. S1H). This pronounced change prompted us to explore the HCC senescent-associated surfaceome (SASome) more broadly by screening ~ 360 well known surface antigens in both HUH7 and HepG2 cells in search for other, potentially directly immunotherapeutically actionable surface antigens (Fig. S2 and S3, respectively). A total of 62 surface antigens showed significant upregulation in HUH7 cells and 50 in HepG2 cells, respectively (Fig. 2A). Out of these 6 or 13 were shared between treatments, whereas others were relatively unique for specific TIS-inducers (Fig. 2A, B), reflecting again TIS heterogeneity at the level of the SASome. Among the more broadly TIS-induced surface antigens, we noted that CD13, CD73, CD146, CD66a and CD54 upregulation for all TIS-inducing treatments in both HUH7 and HepG2 cells (Fig. 2C). Interestingly, expression of these surface markers is associated with an increased tumor invasiveness and metastatic character [24–29]. In line with these findings, we noted a strong upregulation of mesenchymal markers in both cell lines and significant downregulation of CD324/E-cadherin in HUH7 cells (Fig. 2C). Referring back to the RNAseq data, which generally correlated well with surfaceome differences, we could confirm that epithelial-mesenchymal transition (EMT) was among the top 3 enriched hallmark gene sets (Fig. 2D) for both cell lines, independent of the TIS inducer. Other consistently up- or down-regulated hallmark gene sets were coagulation (up), G2M checkpoint and E2F target genes (both down). Upregulation of CD13, CD54, CD66a and CD73 was confirmed by flow cytometry (Fig. S4A) and immunoblot (S4B) and in the former coincided with SA-β-Gal expression (Fig. S4A). Collectively, this indicates TIS in HCC cells appears to directly prompt an induction of metastasis-associated surface antigens and transcriptional pathways.

### HCC therapy-induced senescence induces actionable surface antigens

We also noted consistent induction of CD95, CD276/B7-H3 and CD340 (Fig. 2E), all known targets of therapeutic approaches in cancer. The results from the screen were confirmed via conventional immunoblot (Fig. 2F). Moreover, to investigate co-regulation with the senescent phenotype and verify these findings in additional HCC cell lines, FACS analysis which included a functional SA-β-gal stain, was conducted. These assays verified that increased surface expression of CD95, CD276/B7-H3 and CD340 correlated with SA-β-galactosidase expression, i.e., the senescent phenotype (Fig. 2G). Additionally, the changes observed in the screen for HepG2 and HUH7 could be extended to the other p53-mutated HCC cell lines, HLE and HLF (Fig. S4A). To rule out presence of pre-apoptotic cells, other senescence timepoints were investigated and viability analyses were performed (S4C, S4D, S4E). Moreover, we investigated the expression of these markers in TGCA data for 24 HCC cell lines. Consistent with our observations, high CD276 and CD340 expression was observable, independent of senescence (Fig. 2H). CD95 expression was relatively low but reflected the relative differences between HepG2 (high) and HUH7 (low). A similar picture emerged when 366 cases of primary HCC were analyzed in the TGCA dataset. In keeping with the concept of heterogeneity, this also showed a considerable range of expression between cases. This data from primary cancer samples confirmed that certain SASome-mediated changes are reflected within the range of expression that can be observed upon TIS induction in cell lines. Unexpectedly, even in our screen with cell lines we observed a small subgroup of surface antigens that exhibited bimodality (Table S1), despite the fact that the vast majority of the SASome was regulated uniformly by a given TIS inducer. Among these surface antigens, PD-L1, a key target of immunotherapy [30] was identified (Fig. S4F). Our data thus indicate that TIS in HCC may give rise to SASome heterogeneity with non-responder sub-populations potentially able to escape targeted treatment through bimodal antigen expression.

### TIS-induced surfaceome changes are actionable for apoptosis induction

**Fig. 3:**
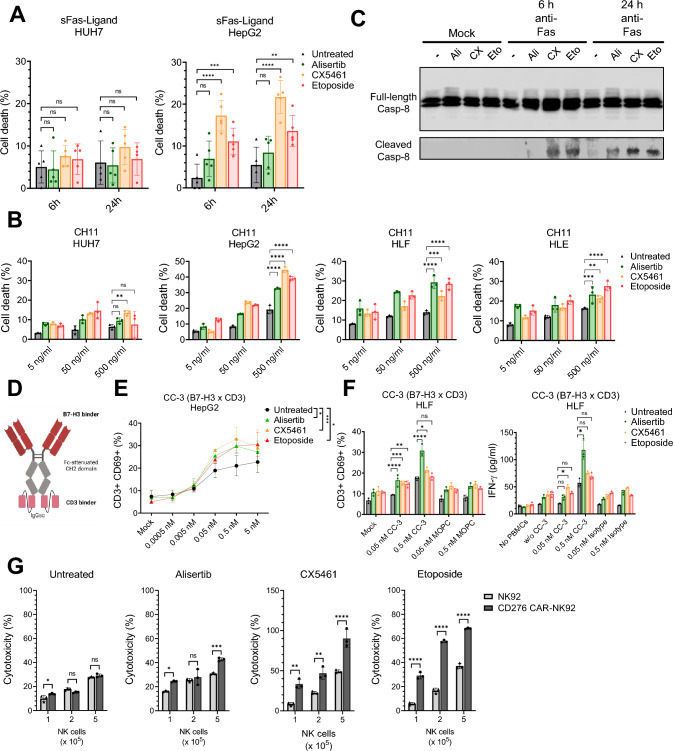
Actionable heterogeneity of hepatocellular carcinoma therapy-induced senescence. **A** Stimulation of senescent HUH7 and HepG2 cells with soluble Fas ligand after senescence-inducing treatment with 1 μM Alisertib (72 h), 500 nM CX5461 (96 h) or 10 μM Etoposide (72 h) (n = 5 biological replicates, combined data, mean + SD, ns *p* > 0.05, *p < 0.05, **p < 0.01, ***p < 0.001, ****p < 0.0001 according to two-way ANOVA). **B** Treatment of senescent HUH7, HepG2, HLF and HLE cells with activating anti-Fas antibody CH11 after senescence-inducing treatment with 1 μM Alisertib (72 h), 500 nM CX5461 (96 h) or 10 μM Etoposide (72 h) (*n* = 3 biological replicates, representative data, ns *p* > 0.05, *p < 0.05, **p < 0.01, ***p < 0.001, ****p < 0.0001 according to two-way ANOVA for highest CH11 concentration). **C** Caspase-8 immunoblot analysis after treatment of senescent HepG2 cells with activating anti-Fas antibody CH11 (*n* = 3 biological replicates, representative data). **D** Schematic overview of T-cell-engaging bispecific B7-H3xCD3 (CC-3) antibody. **E** % of activated CD69 + T cells after co-culture of senescent/non-senescent HepG2 cells with monocyte-depleted PBMCs in presence of T-cell-engaging bispecific antibody CC-3 (*n* = 3 biological replicates, combined data, mean + SD, ns *p* > 0.05, *p < 0.05, **p < 0.01, ***p < 0.001, ****p < 0.0001 according to two-way ANOVA of second highest CC-3 concentration). **F** % of activated CD69^+^ T cells and IFN-γ secretion after co-culture of senescent/non-senescent HLF cells with monocyte-depleted PBMCs in presence of T-cell-engaging bispecific antibody CC-3 (*n* = 3 biological replicates, combined data for surface marker assay, representative data for secretion assay, mean + SD, ns *p* > 0.05, *p < 0.05, **p < 0.01, ***p < 0.001, ****p < 0.0001 according to two-way ANOVA) **(G)** CD276-CAR-NK-92 killing assay of senescent/non-senescent HepG2 cells (*n* = 3 biological replicates, combined data, mean + SD, ns *p* > 0.05, *p < 0.05, **p < 0.01, ***p < 0.001, ****p < 0.0001 according to two-way ANOVA)

While the induction of metastasis-associated hallmarks by TIS is concerning, we speculated whether any of the altered surface markers might represent TIS-induced vulnerabilities that could be amenable to therapeutic targeting. We first focused on the upregulation of CD95 as it might provide ways to circumvent apoptosis resistance frequently seen in cancer senescence [6]. To test whether CD95/Fas expression might re-establish extrinsic apoptosis induction, we treated senescent and non-senescent HUH7 and HepG2 cells with soluble Fas ligand (sFasL) and investigated cytotoxic effects. Strikingly, sFASL-mediated cell death was strongly increased for CX5461- and etoposide-treated HepG2 cells but, as expected, not for HUH7 cells (Fig. 3A). In line with these results, stimulation with the activating Fas antibody CH11 [31] resulted in a significant increase in cell death for all HepG2-, HLE- and HLF-TIS models (Fig. 3B). Furthermore, caspase-8 cleavage upon CH11 stimulation was detected by immunoblot (Fig. 3C). Collectively, our data show that the surfaceome of senescent cells modulated by a specific TIS inducer can be utilized to circumvent apoptosis resistance during HCC senescence by a specific, matched treatment. A similar approach could be envisaged for the upregulated death receptor TRAIL-R2 (Fig. S2, S3).

### TIS-induced surfaceome changes are actionable for CAR-NK cells and T-cell-engaging bispecific antibodies

Another actionable target that was upregulated by senescence induction in 3 out of the 4 tested HCC cell lines is CD276, also known as B7-H3, a member of the B7 costimulatory protein family [32]. Due to the high levels of expression in primary HCC and its induction by multiple TIS inducers, we tested the effect of CC-3 [17], a bispecific CD276-CD3 “T cell engaging” [33] antibody (Fig. 3D). CC-3 was recently shown to induce profound T cell reactivity against various pancreatic, hepatic and gastric cancer cell lines [34]. For this purpose, PBMC were incubated with senescent or non-senescent HepG2 or HLF cells and the % of activated CD69^+^ T cells was determined by flow cytometry. Evidently, the use of the bispecific antibody increased the percentage of activated T cells (Fig. 3E). For HLF cells, alisertib uniquely induced increased T cell activation (as assessed by CD69 upregulation) and IFNγ release correlating with the induction profile of CD276 expression, respectively (Fig. 3F). Of note, there was consistently low T cell activation for non-senescent HCC cells. As a second approach, we explored the effect of TIS-mediated CD276 upregulation on the ability of NK92 cells, fitted with a CD276-specific CAR [35] to kill senescent HepG2 cells. Interestingly, both CX5461 and etoposide, in line with their effect on CD276 upregulation, significantly increased killing rate at all tested NK to target cell ratios for HepG2 cells (Fig. 3G). Collectively, this shows that certain surface antigens induced by specific TIS-inducers might be exploitable by matched antibody- or cell-mediated immunotherapies. The previously addressed inducer-, cell type- and patient specificities of HCC therapy-induced senescence which are a challenge for standardized targeting approaches, may nevertheless be amenable to more individualized approaches that match TIS inducer and immunotherapeutic target.

## Discussion

Previous work has illustrated that senescence may represent a double-edged sword: on the one hand, senescence induction may halt pre-malignant cells and render them susceptible to immune-mediated removal or senolytic immunotherapies; on the other hand, senescent cells represent a reservoir of long-lived cells with oncogenic potential [6, 36]. Our data emphasize this for HCC senescence: TIS induced the expression of metastasis-associated surface markers, that may contribute to tumor invasiveness. At the same time, TIS induced potential vulnerabilities in HCC cells such as CD95, CD276 and CD340 expression. The former two were shown to appear targetable, at least in vitro. While careful exploration for each potential vulnerability will be necessary, the examples provided here support the notion that induction of senescence in combination with a matched senolytic and/or immunotherapeutic approach may be a powerful way to treat HCC, whereas either method alone may increase pro-metastatic features of HCC cells or fail to act on senescent HCC specifically. Of course, metastatic SASome features could also be targeted, but this was not investigated here.

**Fig. 4:**
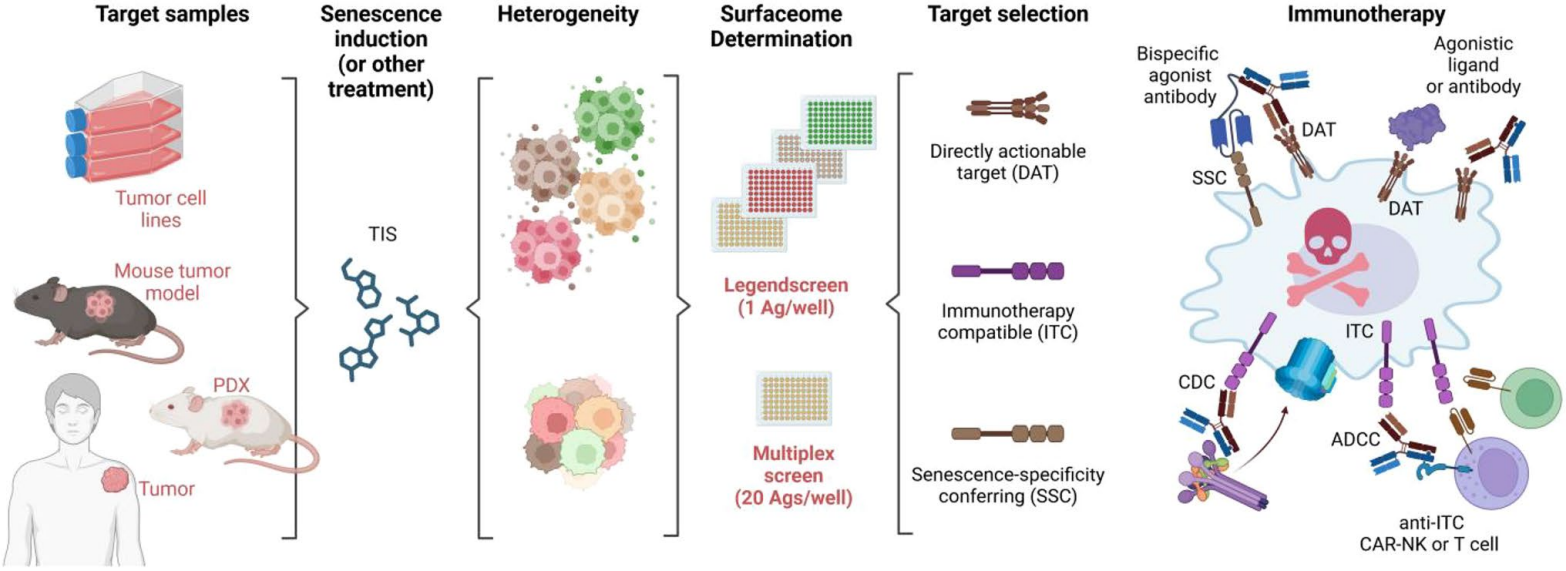
SASome-matched immunotherapy approach. Schematic overview of single-plex or multiplex approach to combine senescence-inducing therapy with conventional surfaceome-based immunotherapy. Identified induced and senescence-specific antigens could be used for direct cell death inductions (DAT) or cytotoxcity via complement (CDC) or NK-/T-cell CARs (ADCC) with potential combination with increased senescence-specificity conferring antigens matched to the respective TIS-inducing therapy

The heterogeneity observed for different TIS regimens suggests that the underlying initiation mechanism may have to be closely matched to a subsequent therapeutic approach. We propose SASome analysis as shown here may be a straightforward approach to identify TIS-specific new actionable targets and would, in principle, be applicable to primary tumor material and/or ex vivo cultures of HCC cells (Fig. 4). On the other hand, the limited range of SASome antigens may be overcome by filtering regulated transcripts from tumor RNAseq data for ‘plasma membrane’ GO terms. Applied to our data this would highlight an additional 70 targets for further study that were upregulated in all cell lines and for at least one treatment but not included in the SASome screen (Fig. S4G, Table S2). Combining transcriptomics and surface analysis for in vitro screening of exposed vulnerabilities might thus help maximize the number of actionable TIS-associated surface structures for appropriate matched immunotherapeutic approaches in future, although this awaits functional testing. In such an analysis, CD95 and CD276 emerged as actionable TIS-induced surface antigens. In isolation, the broad expression of CD95 probably limits its utility as a single agent as indicated by preclinical studies in which hepatitis and other dose-dependent side effects were evidenced [37]. However, in a bispecific antibody format, combining CD95 (senescence-specificity) with another senescent tumor-associated antigen like CD276 or CD340 (to enforce tumor-specificity further) might be able to capitalize on the ~ 20-fold CD95 expression increase and re-establishment of apoptosis in senescent HCC cells. This might allow for a lower dosing regimen that reduces the risk of targeting non-tumorigenic CD95-positive cells and side-effects in general. Given the favorable safety profile of trastuzumab and its efficacy in breast cancer therapy [38, 39] CD340 expression may also be an advantageous single target that could be explored to target TIS HCC cells, but this remains to be verified. Additionally, the expression of CD276 could render senescent cells vulnerable to more sophisticated CAR-based cellular immunotherapies. We recognize that our data in this regard are exclusively limited to an in vitro setting for a small number of targets, and, of course, further pre-clinical work may be required to explore the potential of combining e.g., CX5461 with senolytic targeting approaches that, compared to other cancer entities, are lagging behind for HCC. Nevertheless, we contend ‘SASome-matched immunotherapy approach’ could be useful to select from a growing number of FDA-approved immunotherapies (whether bispecific antibodies or CARs), those that have the highest match with TIS or conventional tumor cell surfaceomes determined straightforwardly by in vitro analysis of cell lines or, ideally, resected primary tumor material. This approach could reveal vulnerabilities that can be targeted directly through immunotherapy, in combination with senescence-specificity conferring antigens that induce cell death or complement- or NK-/T-cell-CAR mediated cytotoxicity to eliminate senescent HCC cells (Fig. 4).

## Supplementary Information

Below is the link to the electronic supplementary material.

Supplementary file1 Supplementary Figure 1: Innate immune responses are differentially affected by senescence and SASP heterogeneity. (A) CXCL10 secretion from (above) and mRNA regulation (below) in senescent HUH7 cells lines analyzed by triplicate ELISA or RNAseq, respectively (n=5 biological replicates, combined data, ns p>0.05 not indicated mean+SD, ns p>0.05 not indicated *p<0.05, **p<0.01, ***p<0.001 ****p<0.0001, according to Kruskal-Wallis test). Normalized RNAseq expression levels relative to the untreated condition are indicated below, with dot areas proportional to fold change. (B, C) Neutrophil migration assessed as shown in (B) and quantified by counting (C) (n=3 biological replicates, combined data, mean+SD, ns p>0.05 not indicated *p<0.05, **p<0.01, ***p<0.001 ****p<0.0001, according to two-way ANOVA). (D) Neutrophil activation assessed by MPO and MMP-9 secretion (n=3 biological replicates, combined data, mean+SD, ns p>0.05 not indicated *p<0.05, **p<0.01, ***p<0.001 ****p<0.0001, according to Kruskal-Wallis and two-way ANOVA) (E, F) NK cell killing of HCC cell lines assessed as shown in (E) and quantified by triplicate LDH release assay. (F) (n=3 biological replicates, representative data, mean+SD, ns p>0.05 not indicated, *p<0.05, **p<0.01, ***p<0.001 ****p<0.0001, according to two-way ANOVA). (G) NK cell killing of senescent IMR90 fibroblasts, quantified by triplicate LDH release assay (n=3 biological replicates, representative data, mean+SD, ns p>0.05 not indicated, *p<0.05, **p<0.01, ***p<0.001 ****p<0.0001, according to two-way ANOVA). (H) ICAM-1 expression histograms of senescent HUH7 cells (n=3, biological replicates representative data). (TIFF 9543 KB)

Supplementary file2 Supplementary figure 2: Surfaceome analysis of senescent HUH7 cell. Data represented as heat-map of logarithmic median expression normalized to untreated expression levels (TIFF 9543 KB)

Supplementary file3 Supplementary figure 3: Surfaceome analysis of senescent HepG2 cell. Data represented as heat-map of logarithmic median expression normalized to untreated expression levels (TIFF 9543 KB)

Supplementary file4 Supplementary figure 4: TIS-induced HCC surfaceome change. (A) Flow cytometric validation of metastatic surface marker and SA-β-Gal expression in HCC cell lines, with reciprocal correlation analysis between SA-β-Gal and surface marker expression (n=3 biological replicates, representative data). (B) Immunoblot validation of metastasis marker expression (n=3 biological replicates, representative data). (C) Expression histograms of CD95, CD276, CD340 and functional SA-β-Gal assay of senescent HCC cell lines HLE and HLF (n=3, biological replicates representative data) (D, E) Representative visualization of viability staining from TIS inducer-treated HCC cell lines HUH7 (D) and HepG2 (E) cells (n=3, biological replicates representative data) (F) Representative visualization of surface marker (PD-L1) displaying bimodal expression. (G) Venn diagram of transcripts from Table S2 that have been assigned the GO Term “plasma membrane” and are upregulated at least 1.5 fold upon 1, 2 or 3 TIS treatments. (TIFF 9499 KB)

Supplementary file5 Supplementary Table S1: Surfaceome fluorescent intensity bimodality scores and cell population fractions. Potentially bimodal antigens (1 < score < 2) are highlighted in orange, and bimodal antigens (score > 2) in red. (XLSX 58 KB)

Supplementary file6 Supplementary Table S2: Plasma membrane genes with substantial, monotonic upregulation in both cell lines across both timepoints under at least one treatment. (XLSX 18 KB)

Supplementary file7 Supplementary Table S3: Antibodies used for fluorescence microscopy and immunoblotting. (XLSX 17 KB)

## Data Availability

Transcriptome data were deposited in the Gene Expression Omnibus under ID [data submission started; ID to be added in proof].

## Abbreviations

CAR: Chimeric antigen receptor
EMT: Epithelial-mesenchymal transition
FDG: Fluorescein-Di-β-D-galactopyranoside
GSEA: Gene set enrichment analysis
HCC: Hepatocellular carcinoma
MFI: Median fluorescent intensity
SASP: Senescence-associated secretory phenotype
SA-β-Gal: Senescence-associated β-galactosidase
SASome: Senescence-associated surfaceome
TIS: Therapy-induced senescence
