# Supplementary figures and images for: Actionable heterogeneity of hepatocellular carcinoma therapy-induced senescence
Source: Cancer Immunol Immunother. 2025 May 15;74(7):207. doi: 10.1007/s00262-025-04060-w (PMC12081809; doi:10.1007/s00262-025-04060-w)

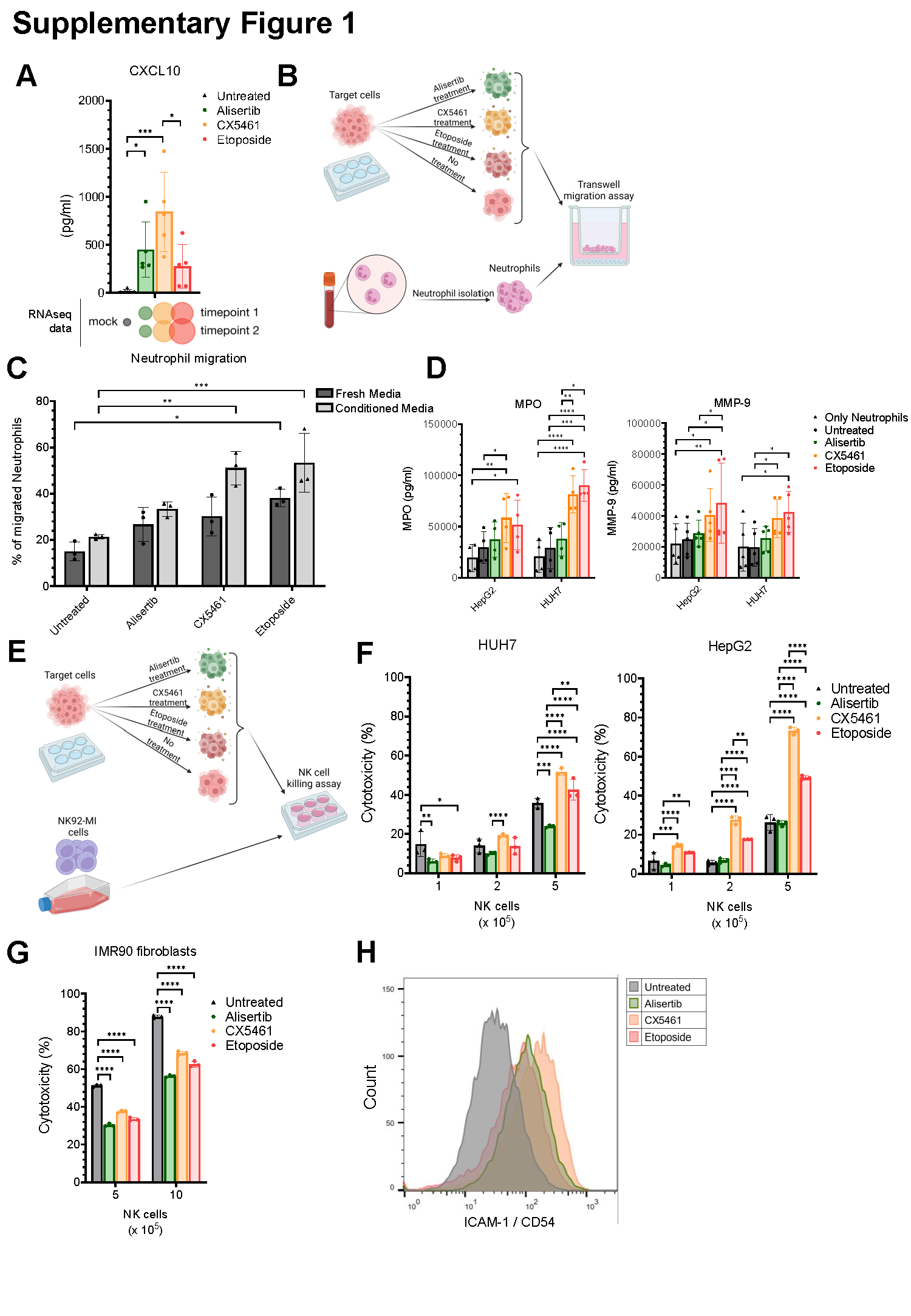

Supplement: Supplementary file 1 — Supplementary file1 Supplementary Figure 1: Innate immune responses are differentially affected by senescence and SASP heterogeneity. (A) CXCL10 secretion from (above) and mRNA regulation (below) in senescent HUH7 cells lines analyzed by triplicate ELISA or RNAseq, respectively (n=5 biological replicates, combined data, ns p>0.05 not indicated mean+SD, ns p>0.05 not indicated *p<0.05, **p<0.01, ***p<0.001 ****p<0.0001, according to Kruskal-Wallis test). Normalized RNAseq expression levels relative to the untreated condition are indicated below, with dot areas proportional to fold change. (B, C) Neutrophil migration assessed as shown in (B) and quantified by counting (C) (n=3 biological replicates, combined data, mean+SD, ns p>0.05 not indicated *p<0.05, **p<0.01, ***p<0.001 ****p<0.0001, according to two-way ANOVA). (D) Neutrophil activation assessed by MPO and MMP-9 secretion (n=3 biological replicates, combined data, mean+SD, ns p>0.05 not indicated *p<0.05, **p<0.01, ***p<0.001 ****p<0.0001, according to Kruskal-Wallis and two-way ANOVA) (E, F) NK cell killing of HCC cell lines assessed as shown in (E) and quantified by triplicate LDH release assay. (F) (n=3 biological replicates, representative data, mean+SD, ns p>0.05 not indicated, *p<0.05, **p<0.01, ***p<0.001 ****p<0.0001, according to two-way ANOVA). (G) NK cell killing of senescent IMR90 fibroblasts, quantified by triplicate LDH release assay (n=3 biological replicates, representative data, mean+SD, ns p>0.05 not indicated, *p<0.05, **p<0.01, ***p<0.001 ****p<0.0001, according to two-way ANOVA). (H) ICAM-1 expression histograms of senescent HUH7 cells (n=3, biological replicates representative data). (TIFF 9543 KB) [file 262_2025_4060_MOESM1_ESM.tiff]

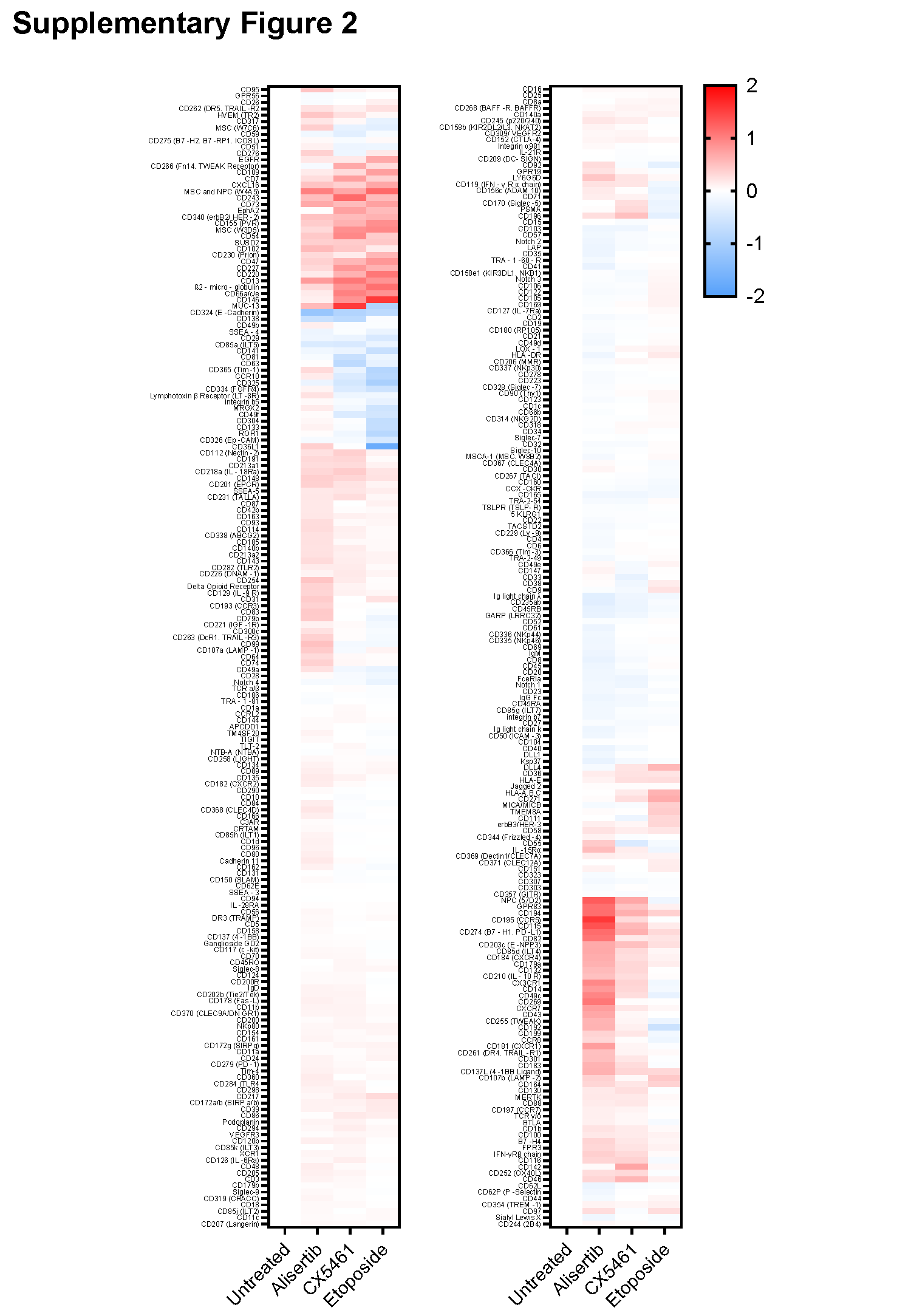

Supplement: Supplementary file 2 — Supplementary file2 Supplementary figure 2: Surfaceome analysis of senescent HUH7 cell. Data represented as heat-map of logarithmic median expression normalized to untreated expression levels (TIFF 9543 KB) [file 262_2025_4060_MOESM2_ESM.tiff]

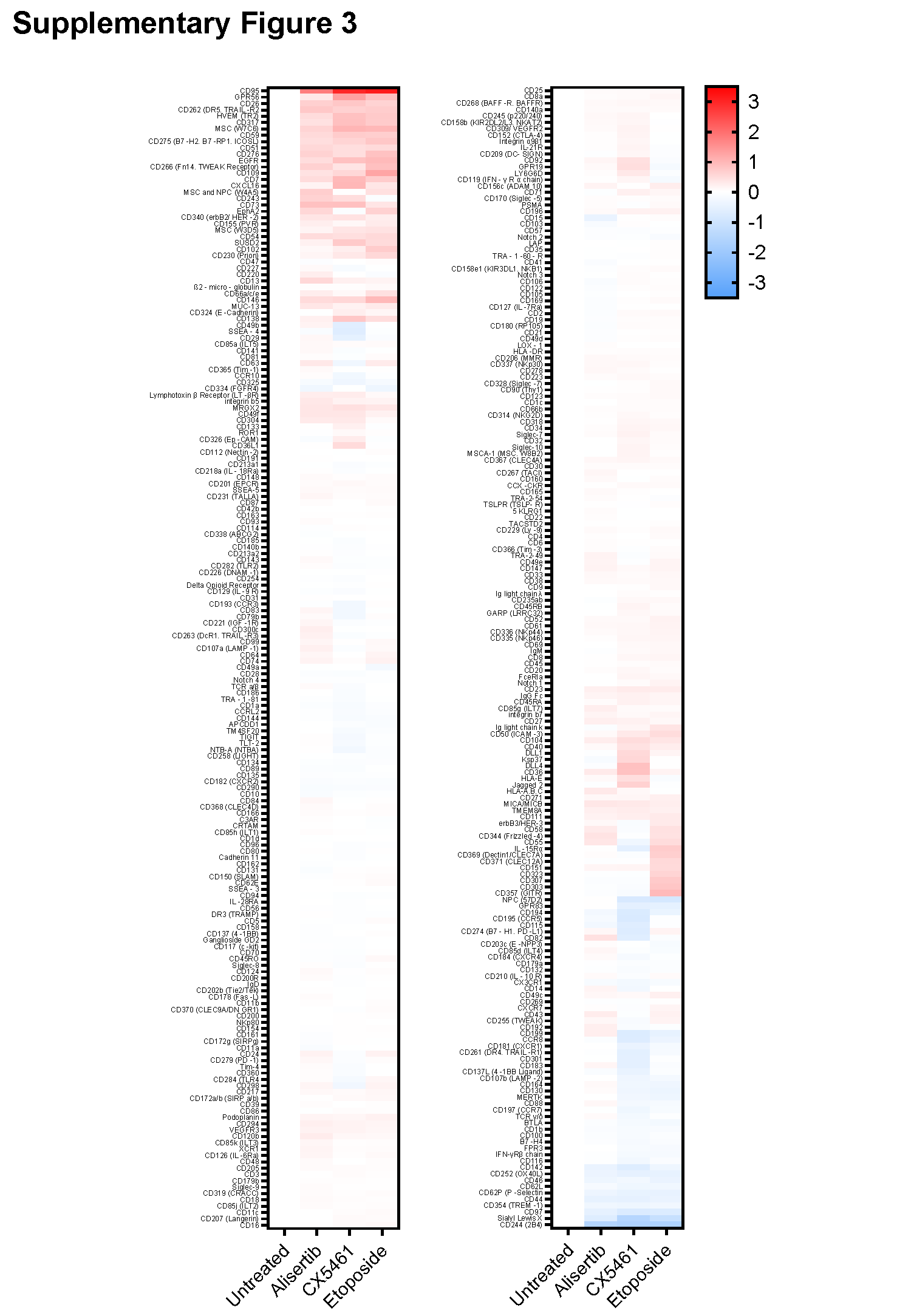

Supplement: Supplementary file 3 — Supplementary file3 Supplementary figure 3: Surfaceome analysis of senescent HepG2 cell. Data represented as heat-map of logarithmic median expression normalized to untreated expression levels (TIFF 9543 KB) [file 262_2025_4060_MOESM3_ESM.tiff]

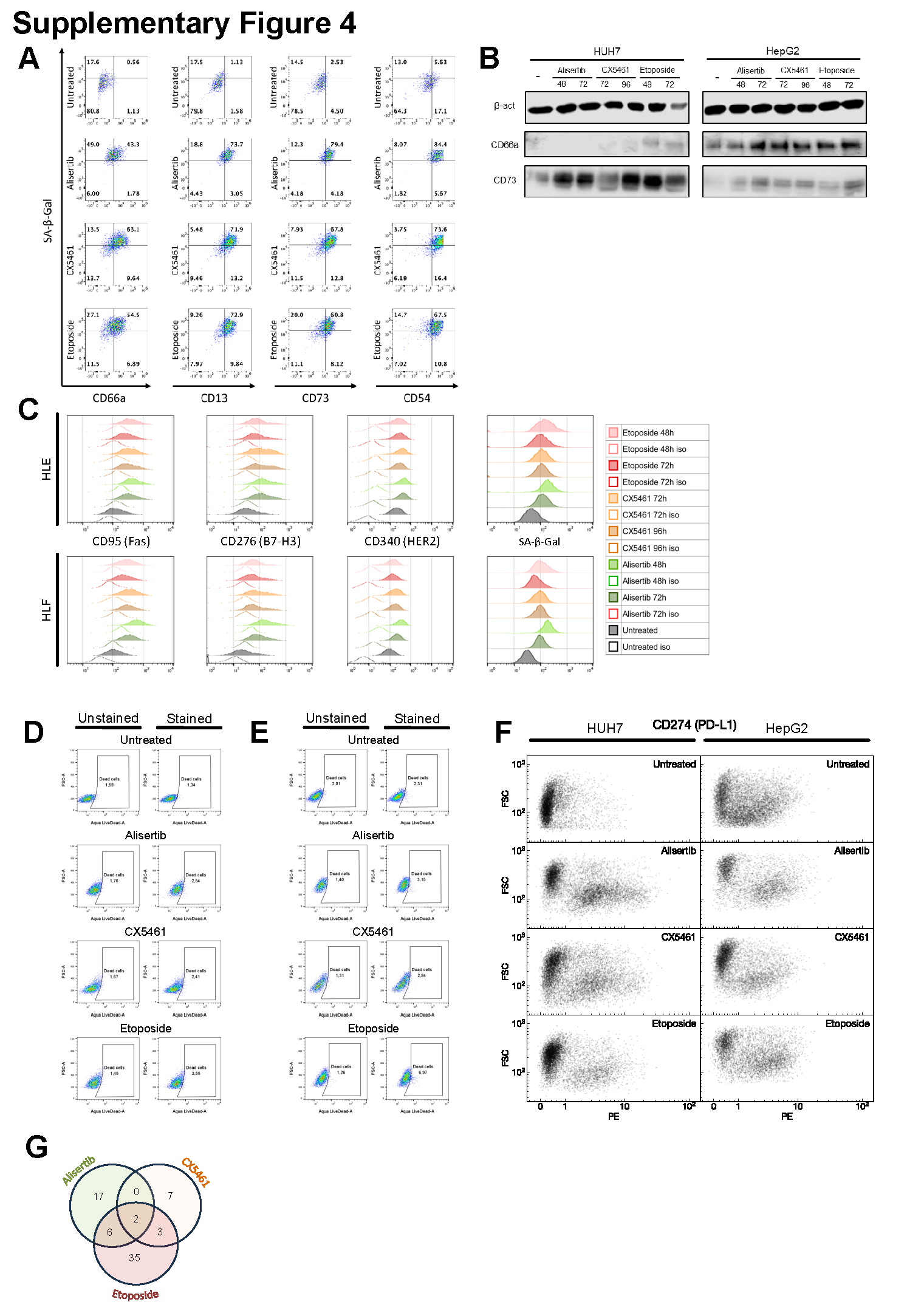

Supplement: Supplementary file 4 — Supplementary file4 Supplementary figure 4: TIS-induced HCC surfaceome change. (A) Flow cytometric validation of metastatic surface marker and SA-β-Gal expression in HCC cell lines, with reciprocal correlation analysis between SA-β-Gal and surface marker expression (n=3 biological replicates, representative data). (B) Immunoblot validation of metastasis marker expression (n=3 biological replicates, representative data). (C) Expression histograms of CD95, CD276, CD340 and functional SA-β-Gal assay of senescent HCC cell lines HLE and HLF (n=3, biological replicates representative data) (D, E) Representative visualization of viability staining from TIS inducer-treated HCC cell lines HUH7 (D) and HepG2 (E) cells (n=3, biological replicates representative data) (F) Representative visualization of surface marker (PD-L1) displaying bimodal expression. (G) Venn diagram of transcripts from Table S2 that have been assigned the GO Term “plasma membrane” and are upregulated at least 1.5 fold upon 1, 2 or 3 TIS treatments. (TIFF 9499 KB) [file 262_2025_4060_MOESM4_ESM.tiff]
